# Robotic Intraoperative Imaging in Orthopaedic and Trauma Surgery: Initial Clinical Experience With a Self‐Driving Mobile 3D C‐Arm in the First 300 Cases

**DOI:** 10.1002/rcs.70195

**Published:** 2026-06-15

**Authors:** Benno Bullert, Luca Ruebel, Jula Gierse, Eric Mandelka, Fenna Brunken, Paul A. Gruetzner, Sven Y. Vetter

**Affiliations:** ^1^ BG Klinik Ludwigshafen, Department for Orthopaedics and Trauma Surgery at Heidelberg University Ludwigshafen Germany

**Keywords:** 3D‐C‐arm, C‐arm repositioning, intraoperative imaging, robotic imaging

## Abstract

**Background:**

Intraoperative C‐arm imaging is essential in orthopaedic and trauma surgery, particularly with the increasing use of minimally invasive techniques. Conventional repositioning is performed by non‐scrubbed staff, while robotic imaging systems are mainly limited to hybrid operating rooms. This study presents the first clinical experience with a fully motorised, self‐driving mobile 3D‐C‐arm.

**Methods:**

In this prospective, single‐center study, 300 procedures were analyzed using device log data and perioperative parameters to assess imaging workflow and system performance.

**Results:**

Of 300 procedures, 279 were included. Mean procedure time was 104.7 ± 57.4 min, with a C‐arm operation time of 31.9 ± 29.1 min, corresponding to a C‐arm operation ratio (COR) of 35.2%. Automated positioning accounted for 30.7% of movements, and 37.9% of images were acquired from stored positions.

**Conclusion:**

The system enabled sterile‐field control and demonstrated potential to support intraoperative imaging workflow. Further comparative studies are required to evaluate its clinical impact.

## 1 Introduction

Intraoperative imaging using C‐arms is a standard procedure for evaluating operative results in orthopaedic and trauma surgery. The growing number of minimally invasive approaches has also increased the importance of intraoperative imaging [1, 2].

The repetitive positioning of the C‐arm during surgery has generally been the task of non‐scrubbed operating room (OR) staff, including C‐arm technicians [3]. Various studies have therefore been carried out to make this repositioning more precise and effective [2, 3, 4, 5, 6, 7, 8]. The motorization of the C‐arm's rotational axes [9] and the driving platform [10] has already been tested in experimental settings.

In imaging, the last quantum leap was the switch to flat‐panel detector technology [11]. Robotic, motorised imaging systems are currently mainly available as hybrid operating rooms (hybrid ORs). However, the availability of such hybrid ORs is still very limited, which is why their use in orthopaedic and trauma surgery is mostly reserved for complex pelvic and spine interventions involving navigated procedures [12, 13]. Nevertheless, the use by orthopaedic and trauma surgeons remains low [14].

Meanwhile, the benefits of these hybrid ORs are controversial. Some studies show advantages, such as independent C‐arm operation by the surgeon and reduced unintentional ‘fluoro hunting’ [12, 13], while others show disadvantages, such as complex setup and prolonged procedure time [14].

In addition to hybrid ORs, mobile motorised imaging systems are now available. As a mobile imaging system, the Loop‐X (Brainlab AG, Munich, Germany) can save positions and return to them fully motorised [15, 16]. Another mobile motorised imaging system is the Excelsius3D C‐arm (Globus Medical Inc., Audubon, PA, USA).

The C‐arm investigated in this study is also a fully motorised, self‐driving mobile 3D‐C‐arm (Ciartic Move, Siemens Healthineers AG, Forchheim, Germany). It enables the surgeon to completely control intraoperative mobile C‐arm imaging from the sterile field. In addition, positions of the C‐arm can be saved and accessed repeatedly. This means that time‐consuming intraoperative repositioning can be automated.

The definition of a hybrid OR was determined by a committee of experts [17]. With its combination of 3D imaging, fluoroscopy, and sterile hand control, the motorised mobile 3D‐C‐arm in this study meets all the criteria for a hybrid OR while retaining the mobility of a simple C‐arm.

This study aims to show the first clinical experience with a fully motorised, self‐driving mobile 3D‐C‐arm in orthopaedic and trauma surgery and to present a comprehensive quantitative analysis of intraoperative imaging. For this purpose, intraoperative data from the first 300 procedures were systematically recorded and analyzed with respect to procedural and device‐specific parameters.

## 2 Material and Methods

The study was approved by the ethics committee of the responsible state medical association (application number 2023‐17252). All procedures were in accordance with the ethical standards of the institutional and national research committee and with the 1964 Helsinki declaration and its later amendments or comparable ethical standards.

All persons involved in the use of the 3D‐C‐arm were instructed in its use during an initial 2‐h training session.

### 2.1 Description of the Fully Motorised, Self‐Driving Mobile 3D‐C‐Arm

**FIGURE 1:**
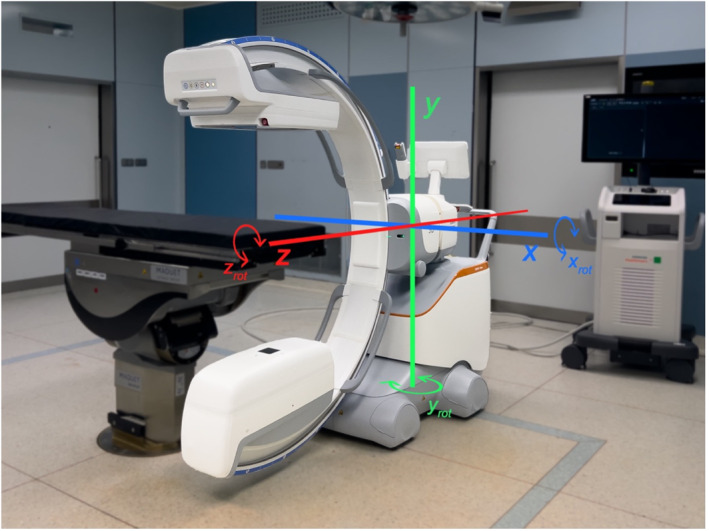
Motorised DoF (degrees of freedom) of the 3D‐C‐arm. Three translational DoF (*x; y; z*) and three rotational DoF (*x*_rot_; *y*_rot_; *z*_rot_).

The 3D‐C‐arm has motorised holonomic wheels that enable it to move and rotate in the horizontal plane (translational movement along the *x*‐ and *z*‐axes, and *y*‐rotation) within a confined space. The lifting column enables motorised vertical movement (translational movement along the *y*‐axis). The ‘C’ is also motorised, enabling orbital rotation and angulation (*x*‐ and *z*‐rotation). The 3D‐C‐arm therefore has 6 motorised degrees of freedom (DoF), divided into 3 translational DoF and 3 rotational DoF (Figure 1).

The 3D‐C‐arm can store 12 positions (5 on the wireless hand control) that can be accessed automatically if required. The individual collimation and image rotation information is also stored at each position and automatically adjusted when the desired position is reached.

The 3D‐C‐arm has a wireless hand control and a remote touch monitor for control purposes. Both can be covered with sterile drapes and are therefore available to the sterile staff at the operating table, allowing the 3D‐C‐arm to be operated completely autonomously by the sterile staff.

Alternatively, the 3D‐C‐arm can be controlled by the C‐arm‐tech on the handle or by the wired hand control.

### 2.2 Definitions of Terms

To describe and analyze the imaging process, various parameters were defined as follows:

Procedural:

Operation: An operation includes requesting the C‐arm from the parking position into the surgical site, imaging, and then returning to the parking position.
C‐arm Operation Time (COT): Time during which the C‐arm is used in an operation.
C‐arm Operation Ratio (COR): COT/procedure time, shows the proportion of time the C‐arm is used in relation to the procedure time.

Automation:

Stored Position Movement (SPM): Number of movements that are automated based on the position storage function.
Stored Position Ratio (SPR): Proportion of SPM in all movements of a procedure.
Image Position Ratio (IPR): Proportion of 2D images in all 2D images of a procedure that were created exactly in one of the stored positions.

### 2.3 Log Files

To acquire data on the intraoperative use of the 3D‐C‐arm, the log files of the 3D‐C‐arm were analyzed postoperatively. These contain the following data, which was collected for the individual procedures:

— Movement information of all motorised movement axes
— Position information of orbital rotation and angulation
— Position storage information of the C‐arm (storing, approaching, reaching)
— Radiation information, including radiation triggering, radiation duration, imaging type, fluoroscopy time, and dose‐area product (DAP)

### 2.4 Data Collection

To provide a structured description of intraoperative imaging workflow, prospectively collected perioperative data were combined with device log file data, allowing assessment of procedural timing, frequency of C‐arm use, automated repositioning, and imaging‐related parameters.

The following data were collected prospectively:

1. Operated anatomies
2. Procedural parameters
  - Procedure time
  - C‐Arm Operating Time (COT)
  - C‐Arm Operating Ratio (COR)
  - Operations
  - Preoperative Operations
3. Automation
  - Stored Position Movement (SPM)
  - Stored Position Ratio (SPR)
  - Image Position Ratio (IPR)
4. Imaging
  - 2D images
  - 3D scans
  - Fluoro‐Time
  - Dose‐Area‐Product (DAP)

For analysis, the data were differentiated by the operated anatomy and body area.

### 2.5 Statistics

Statistical analyses were performed with IBM SPSS Statistics Version 29 (IBM Corp., Armonk, NY, USA). Quantitative data are shown as mean ± standard deviation (SD). The correlation was calculated according to Pearson. A significance level of *p* < 0.05 was set.

## 3 Results

### 3.1 Included Procedures

The first 300 cases with the fully motorised, self‐driving mobile 3D‐C‐arm were included in the study. The log files, which served as the basis for the analysis, were available for 287 of these cases.

A further 8 cases were excluded from the analysis for various reasons (malfunction, used by another department), resulting in an analysis of 279 included cases.

### 3.2 Procedures

**FIGURE 2:**
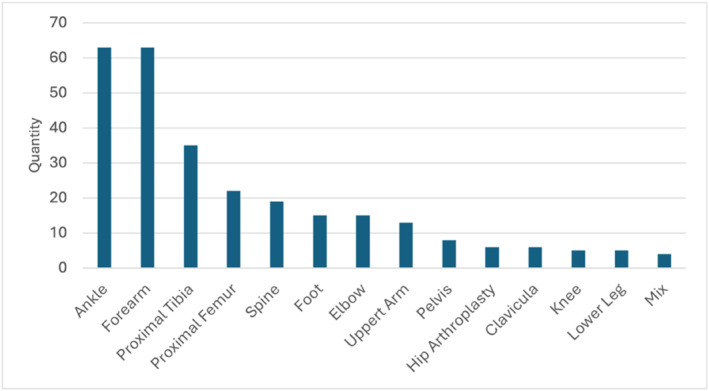
Procedures by anatomy.

A total of *n* = 151 cases (54.1%) were attributable to the lower extremity, with the majority of interventions on the ankle joint (*n* = 63). Followed by the upper extremity with *n* = 91 cases, with a noticeable majority of cases on the forearm (*n* = 63). *n* = 33 procedures were performed on the trunk, *n* = 4 cases were of mixed anatomy. All procedures are listed by anatomy (Figure 2) and by areas (Table 1).

**TABLE 1:** Procedures by area.

	*n*	%
Trunk	33	11.8%
Upper extremity	91	32.6%
Lower extremity	151	54.1%
Mix	4	1.4%

### 3.3 Procedural Parameters

Across all cases, the average procedure time was 104.72 ± 57.37 min. With an average COT of 31.86 ± 29.12 min, the C‐arm was in operation for 35.2% ± 26.8% (COR) of the procedure time.

The two anatomies associated with the highest C‐arm utilisation were proximal femur (COR = 69.6% ± 23.1%) and spine (COR = 47.1% ± 34.4%). The lowest C‐arm utilisation was observed in hip arthroplasty (COR = 8.7% ± 2.0%) and pelvic surgery (COR = 11.8% ± 4.7%).

**FIGURE 3:**
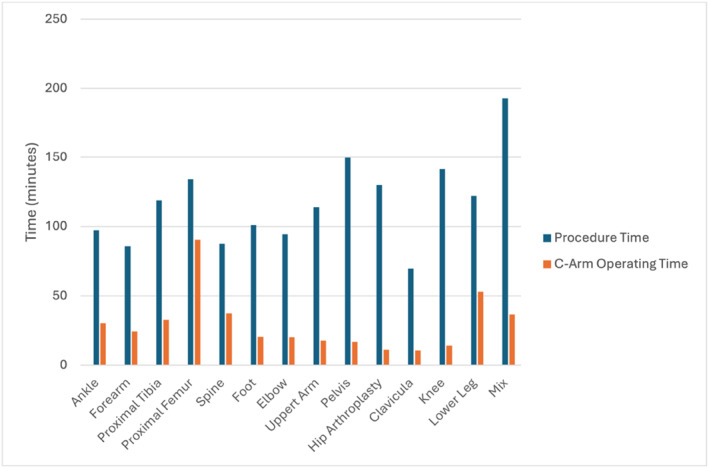
Procedure time and C‐arm operating time.

A detailed view of all procedure times and C‐arm operating times can be found in Figure 3.

### 3.4 Operations

Overall, the C‐arm was used for an average of 4.16 ± 2.28 operations per surgery. Procedures on the extremities (upper extremity: 4.62 ± 2.58; lower extremity: 3.95 ± 2.15) show a significantly higher number of operations of the C‐arm than procedures on the trunk (3.58 ± 1.62) (*p* = 0.041).

Preoperative operations were more frequently used for procedures on the trunk. In 89.5% of all spinal procedures, an imaging operation was performed before the skin incision, followed by procedures on the proximal femur (81.8%) and the pelvis (75.0%) (Table 2).

**TABLE 2:** Operations and preoperative operations.

		Operations		Preoperative operations	
		Mean	SD	*n*	Ratio (%)
Overall		4.16	2.28		
Trunk		3.58	1.62		
	Spine	3.26	1.59	17	89.5
	Pelvis	4.88	1.36	6	75.0
	Clavicula	2.83	1.17	2	33.3
Upper extremity		4.62	2.58		
	Forearm	4.84	2.59	8	12.7
	Elbow	3.53	1.68	1	6.7
	Upper arm	4.77	3.19	2	15.4
Lower extremity		3.95	2.15		
	Ankle	4.44	2.45	11	17.2
	Proximal tibia	4.31	2.23	4	11.4
	Proximal femur	2.86	1.25	18	81.8
	Foot	3.53	1.25	0	0.0
	Hip arthroplasty	2.50	0.55	0	0.0
	Knee	2.40	1.34	0	0.0
	Lower leg	4.60	1.52	1	20.0
Mix		6.75	2.06	0	0.0

### 3.5 Position Storage

A total of almost 1/3 of the movements of the C‐arm were realised by stored position movements (SPR = 30.7% ± 21.7%). At the same time, over 1/3 of all images were captured in stored positions (IPR = 37.9% ± 28.5%) (Table 3).

**TABLE 3:** Position storage.

		Mean	SD
Overall	Stored position movement (SPM)	7.41	7.07
	Stored position ratio (SPR)	30.7%	21.7%
	Image position ratio (IPR)	37.9%	28.5%
Trunk	SPM	8.73	5.90
	SPR	35.9%	16.8%
	IPR	44.6%	24.2%
Upper extremity	SPM	6.15	5.90
	SPR	34.2%	25.0%
	IPR	37.8%	32.6%
Lower extremity	SPM	7.88	8.00
	SPR	27.6%	19.9%
	IPR	37.1%	26.7%
Mix	SPM	7.00	6.22
	SPR	25.8%	27.9%
	IPR	14.8%	9.9%

### 3.6 Imaging

On average, the highest number of images was obtained in lower extremity procedures (60.05 ± 72.37), while the lowest average number of images per procedure was obtained in upper extremity procedures (40.49 ± 28.13).

Dose area product (DAP) was highest at the trunk (5392.3 ± 7727.2 mGy × cm^2^) and lowest at the upper extremity (801.5 ± 943.2 mGy × cm^2^). Fluoro time, in contrast, was lowest at the trunk (72.4 ± 67.9 s) and highest at the lower extremity (165.1 ± 125.6 s) (Table 4).

**TABLE 4:** Imaging parameters.

		Mean	SD
Trunk	2D‐image	53.06	51.54
	3D‐scan	0.45	0.83
	Fluoro‐time	72.4 s	67.9 s
	DAP	5392.3 mGy × cm^2^	7727.2 mGy × cm^2^
Upper extremity	2D‐image	40.49	28.13
	3D‐scan	0.32	0.61
	Fluoro‐time	151.9 s	102.8 s
	DAP	801.5 mGy × cm^2^	943.2 mGy × cm^2^
Lower extremity	2D‐image	60.05	72.37
	3D‐scan	1.19	1.20
	Fluoro‐time	165.1 s	125.6 s
	DAP	2829.3 mGy × cm^2^	3551.1 mGy × cm^2^
Mix	2D‐image	47.75	22.01
	3D‐scan	1.00	0.82
	Fluoro‐time	194.0 s	120.3 s
	DAP	1396.2 mGy × cm^2^	1217.6 mGy × cm^2^

## 4 Discussion

The study aims to present the initial clinical experience with a fully motorised, self‐driving mobile 3D‐C‐arm. At the same time, the data collection provides the opportunity to record detailed quantitative data on intraoperative imaging in trauma surgery.

To our knowledge, there are currently no further studies on the application and evaluation of such an imaging device. Nor are we currently aware of any studies that provide a detailed quantitative analysis of intraoperative imaging in general.

In the first 300 cases, mainly lower extremity surgeries were performed, followed by surgeries on the upper extremity and the trunk.

Each staff member received 2 h of training from the manufacturer before using the system. Since both surgeons and OR staff were already trained in the use of intraoperative 2D and 3D imaging with other systems, the new 3D‐C‐arm could be implemented quickly and safely after initial training.

In all cases included, the system was operated by staff at the operating table using the sterile‐draped wireless hand control. Support from the non‐sterile OR team was required only to adjust the collimation and image rotation. These functions could have also been carried out by the OR staff using the sterile remote touch monitor. However, the remote touch monitor was not used in the first few cases.

### 4.1 C‐Arm Operation Time

The measurement of C‐arm operating time (COT) represents the total time the C‐arm is in use during the surgical procedure. Because this parameter is largely dependent on procedure time, the ratio of COT to procedure time (COR) was determined. This provides a parameter that is comparable between different procedures and indicates the extent to which the C‐arm is used during a procedure.

Overall, the average COR for the first 300 cases is 35.2%. This high percentage underscores the importance of intraoperative imaging in trauma surgery. At the same time, this ratio is an indicator of the great potential that optimisation of intraoperative imaging has on the surgical process.

Among the different anatomical areas, imaging plays the greatest role in the lower extremity (COR = 38.5%). A more detailed analysis shows that interventions on the proximal femur are associated with the highest amount of intraoperative imaging (COR = 69.6%). In our experience, smaller C‐arms that are easier to manoeuvre have tended to be used for the treatment of proximal femur fractures. The projection changes with these smaller detectors are already very challenging due to the many axes that must be adjusted in parallel. Intraoperatively, however, they have to be performed several times [2]. The use of the fully motorised mobile 3D‐C‐arm may help to reduce several difficulties commonly associated with manual imaging, particularly during repeated projection changes and return to previously defined imaging positions. Due to a larger field of view, the detector can have a greater distance to the surgical field [11] while visualizing the complete region of interest on the proximal femur is possible at the same time. This eliminates the need for smaller movements to obtain a better view of the entire surgical field. Furthermore, thanks to its larger gantry, the C‐arm can remain at the table almost continuously, which may support rapid image acquisition when needed.

### 4.2 Operations

Once the parking and table positions have been defined, the fully motorised 3D‐C‐arm can move to these positions repetitively and automatically by using the sterile hand control. The number of operations, that is, how often the device is requested from the parking position, is an important parameter for analyzing the intraoperative use of the 3D‐C‐arm.

On average, 4.16 operations per procedure were observed across all cases. A detailed look at the different anatomical areas shows a slightly lower number of operations on the trunk compared to the extremities. This might be due to fewer individual operations required for spine cases, especially in conventional dorsal instrumentation. However, in these spine cases, the individual operations take considerably longer. Therefore, a continuous use of intraoperative imaging can be observed rather than a division into individual operations.

A similar pattern can be seen in the use of the C‐arm at the proximal femur. Again, there is a low average number of operations (2.86), but a high C‐arm operation ratio (COR). This indicates long single operations, which means continuous use of the C‐arm in the surgical procedure.

The number of preoperative operations shows that imaging is already regularly used before skin incision, especially for procedures on the spine, pelvis, and proximal femur. In the context of the fully motorised 3D‐C‐arm, this means that important positions can already be stored at this point, so that the C‐arm can be moved faster to these positions during the procedure.

### 4.3 Position Storage

The analysis of position storage shows that, on average, 30.7% of all intraoperative movements were realised by stored position movements. Furthermore, 37.9% of all images were acquired in stored positions. There is a significant correlation between SPR and the number of operations (*p* < 0.001), indicating greater reliance on position movements in procedures with many operations. This indicates that the surgeons found it especially useful to use automated movements to stored positions for procedures with many operations.

Although the distance of individual movements was not specifically collected, the analysis of the log files shows that mainly long movements, such as the movement from the parking position to the sterile operating field and back, as well as large changes between main projections, were performed by automated positioning movements.

As already mentioned in the introduction, there are two other motorised mobile imaging devices (Loop‐X and Excelsius3D) that can store positions. However, a lack of scientific evaluation of these two devices prevents a comparison at the time of writing this study. Nevertheless, both devices focus on spine surgery due to the close integration of robotic and navigation systems.

### 4.4 Limitations

Many of the measurements still show a large standard deviation, indicating that the individual procedures should be further broken down into more specific interventions. Accordingly, a high number of documented procedures is necessary for a sufficient number of cases in the individual interventions.

As this is the first data from a fully motorised mobile imaging system, a learning curve in using specialized functions, such as position storage, cannot be ruled out. A formal temporal analysis of this potential learning curve was not performed due to the heterogeneity of procedures, surgeons, and operating room teams.

Another limitation is the non‐comparative, single‐center design of the study. As no control group using conventional C‐arm imaging was included, the present study does not allow conclusions regarding comparative workflow efficiency, radiation exposure, or clinical outcomes. In addition, potential selection bias cannot be excluded, as the use of the system may have been influenced by clinical indication, system availability, surgeon preference, and institutional workflow. Therefore, the generalizability of the findings to other clinical settings, institutions, and surgical workflows may be limited.

## 5 Conclusion

The fully motorised, self‐driving mobile 3D‐C‐arm demonstrated potential to support intraoperative imaging in orthopaedic and trauma surgery. Its ability to be operated from the sterile field may enhance workflow efficiency and potentially reduce the need for non‐scrubbed staff intervention. The high proportion of intraoperative imaging within the total procedure time underscores its relevance and illustrates the potential to optimize and streamline surgical processes. However, as this study reflects an initial, non‐comparative clinical experience, further comparative studies are required to evaluate its impact on workflow efficiency, radiation exposure, and clinical outcomes.

## Author Contributions

Benno Bullert contributed to the conception and design of the work, as well as the acquisition, analysis, and interpretation of data. Luca Ruebel was responsible for the acquisition and analysis of data and substantially revised the manuscript. Jula Gierse contributed to the design of the work and the acquisition of data. Eric Mandelka was involved in the conception of the work and substantially revised the manuscript. Paul A. Gruetzner contributed to the conception of the work. Sven Y. Vetter contributed to the conception of the work, revised the manuscript, and participated in data analysis.

## Funding

The authors have nothing to report.

## Ethics Statement

This study was reviewed and approved by the responsible Ethics Committee (Application No. 2023‐17252). To ensure the confidentiality and privacy of the included patients, all data have been anonymized to the extent that no conclusions can be drawn about individual patients. Due to the complete anonymization of the data, individual patient consent and disclosure are not required for this study.

## Consent

The study was approved by the ethics committee of the responsible state medical association (application number 2023‐17252). After a thorough review, the ethics committee determined that individual patient informed consent was not necessary for this study. The rationale provided was that only patients who were already clinically indicated for surgical therapy with C‐arm imaging were included, ensuring that there was no additional risk or radiation exposure for participants. The application of radiation was conducted solely with the justified indication of a qualified physician, in compliance with 83 StrlSchG. The same radiation exposure (type, scope, and frequency) would occur regardless of participation in the study.

During surgery, data were collected solely through perioperative observation. The study had no influence on the surgical procedure or the intraoperative imaging measures.

## Conflicts of Interest

The research group MINTOS had grants/grants pending and technical support from Siemens Healthineers AG (Erlangen, Germany) and Globus Medical Inc. (Audubon, Pennsylvania, USA). The funders had no involvement in the study conceptualization, design, data collection, analysis, nor the decision to publish or the preparation of the manuscript.

## Data Availability

The datasets generated during and/or analysed during the current study are available from the corresponding author on reasonable request.
